# Metabolomic and Proteomic Analysis of Maize Embryonic Callus induced from immature embryo

**DOI:** 10.1038/s41598-017-01280-8

**Published:** 2017-04-21

**Authors:** Fei Ge, Hongmei Hu, Xing Huang, Yanling Zhang, Yanli Wang, Zhaoling Li, Chaoying Zou, Huanwei Peng, Lujiang Li, Shibin Gao, Guangtang Pan, Yaou Shen

**Affiliations:** 1 Key Laboratory of Biology and Genetic Improvement of Maize in Southwest Region, Maize Research Institute, Sichuan Agricultural University, Chengdu, 611130 China; 2 Institute of Animal Nutrition, Sichuan Agricultural University, Chengdu, 611130 China

## Abstract

The low ratio of embryonic callus (EC) induction has inhibited the rapid development of maize genetic engineering. Still, little is known to explain the genotype-dependence of EC induction. Here, we performed a large-scale, quantitative analysis of the maize EC metabolome and proteome at three typical induction stages in two inbred lines with a range of EC induction capabilities. Comparison of the metabolomes and proteomes suggests that the differential molecular responses begin at an early stage of development and continue throughout the process of EC formation. The two inbred lines show different responses under various conditions, such as metal ion binding, cell enlargement, stem cell formation, meristematic activity maintenance, somatic embryogenesis, cell wall synthesis, and hormone signal transduction. Furthermore, the differences in hormone (auxin, cytokinin, gibberellin, salicylic acid, jasmonic acid, brassinosteroid and ethylene) synthesis and transduction ability could partially explain the higher EC induction ratio in the inbred line 18-599R. During EC formation, repression of the “histone deacetylase 2 and ERF transcription factors” complex in 18-599R activated the expression of downstream genes, which further promoted EC induction. Together, our data provide new insights into the molecular regulatory mechanism responsible for efficient EC induction in maize.

## Introduction

All plants possess the capacity of cellular totipotency, as single cells or tissues can regenerate into whole plants through somatic embryogenesis in response to certain stimuli^1^, such as wounding or hormones. Based on cellular totipotency, transformation techniques have been developed for genetic engineering of plants. For many plant species, embryonic callus (EC) is the best tissue for genetic transformation. However, EC induction and plant regeneration are affected by many factors, including hormones, genotypes and the concentrations of various substances in the induction medium^2, 3^.

To date, several studies focused on gene functions products in EC induction or regeneration using proteomic analysis in different plant species. Cellular metabolic process-related proteins and hormone-related proteins were differentially expressed during the process of rice callus differentiation^4^. In addition, carbohydrate metabolism- and glycolysis-related proteins played a role in rice callus differentiation^5^. Furthermore, alpha-amylase was reported to be one of the most important enzymes for somatic embryogenesis^3, 5^. In the process of *Vanilla planifolia* EC induction, the differentially expressed proteins were mostly involved in the function of amino acid-protein metabolism, photosynthetic activity, defense and stress response, and iron storage^6, 7^. Similarly, during *Vitis vinifera* EC induction, oxidative stress response was also activated^8^.

Maize (*Zea mays* L.) is one of the most important staple crops in the world. However, conventional maize genetic breeding is time-consuming and limited by natural variation. Maize genetic transformation is an important approach to circumvent these limitations, which requires the induction of EC prior to the introduction of gene constructs. However, the low EC induction rate for the majority of inbred maize lines requires extensive backcrossing after transformation of the few lines with high EC induction rates. Recently, two studies have reported proteomic changes during EC formation^9^ and somatic embryogenesis^10^. However, they both used two-dimensional electrophoresis (2-DE) combined with mass spectrometry methods, which have several deficiencies: low protein identification ratios, difficulties in quantifying differentially expressed proteins and low reproducibility^11^. Furthermore, each study relied on one inbred line (with a high EC induction capability), A19^9^ or H99^10^, respectively, and was restricted to protein expression changes after EC formation or upon somatic embryogenesis.

Moreover, to account for the genotype dependence of EC induction rates^12^, this study combined iTRAQ-based quantitative proteomics and liquid chromatography mass spectrometry (LC-MS) detected metabolomics to reveal the dynamic and complex network of maize EC formation using the 18-599R inbred line (with a strong capacity of EC formation) and the B73 inbred line (with a low capacity of EC formation).

## Results

### Metabolomic Changes during EC Formation

**Figure 1:**
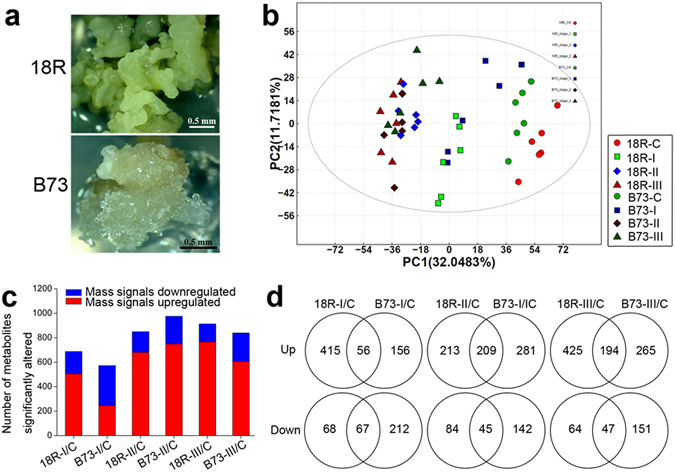
Overview of the maize callus and the metabolomic responses to induction medium in 18R and B73. (**a**) Photos of a single callus of 18R and B73 induced for 15 d. (**b**) Principal component analysis based on metabolite profiles of maize 18R and B73 inbred lines in 3 stages. “18R”: 18R; “C”: control; “I”: stage I; “II”: stage II; “III”: stage III. (**c**) Numbers of individual mass spectrometry features that were significantly changed (fold change >1.5, *Q value* < 0.05) at each stage in each line. (**d**) Venn diagram of significantly up- and down-regulated m/z in 18R and B73 at each stage. “I/C”: stage I relative to control, as well as “II/C,” “III/C.”

Based on morphological feature, the process of embryonic callus formation was divided into embryo expansion (stage I, 1–5 d), initial callus formation (stage II, 6–10 d) and embryonic callus generation (stage III, 11–15 d)^13^. The EC induction ratio of inbred line 18-599R (18R) was high up to 80%^13^, whereas B73 embryos failed to form EC (Fig. 1a). To better understand the metabolite differences during EC formation, total metabolites of control (C), stage I, stage II and stage III were extracted from calli induced for 0 d, 1–5 d, 6–10 d and 11–15 d, respectively. They were then submitted to untargeted high performance liquid chromatography-mass spectrometry (HPLC-MS, biologically replicated six times) analysis. After Loess of signal correction (LSC), the m/z with a relative standard deviation (RSD) between 0 to 30% was submitted to principal component analysis (PCA, Fig. 1b). A separate PCA for different samples showed that the CK, stage I, stage II and stage III differed from one another, as did the samples from 18R and B73. Further partial least-squares discriminant analysis (PLS-DA) showed that, except for 18R stage II and B73 stage II; the samples were distinct from each other (Supplementary Fig. S1). Relative to the control, a total of 1,702 mass (m/z) signals with 1.5-fold changes (Q value < 0.05) in at least one stage in any line were kept (Supplementary Table S1). As shown in Fig. 1c, metabolites up-regulation were more common than down-regulation at each stage for both lines. In addition, at each stage of 18R EC induction, more than 70% of significantly up- and down-regulated mass signals were different from B73 (Fig. 1d). These results suggested that the metabolite changes during EC induction were significantly different between 18R and B73.

We then performed KEGG pathway analysis for all of the differentially altered m/z (http://www.genome.jp/kegg/). Most of the m/z signals belonged to amino acid metabolism and metabolism of other amino acids pathways. Among them, 56 metabolites were assigned to arginine and proline metabolism (Supplementary Table S2). It is well known that proline plays an important role in stress response. To some extent, immature embryos cultured on induction medium just undergo a type of stress (hormone stress). Moreover, 208 metabolites were annotated in carbohydrate metabolism including starch and sucrose metabolism, pentose and glucuronate interconversions, galactose metabolism and fructose and mannose metabolism. In addition, more than 170 metabolites were related to the lipid metabolism pathway (Supplementary Table S2). These results suggested that energy metabolism played an extremely important role in EC induction. Additionally, 50 metabolites related to signal transduction pathways were also differentially expressed between 18R and B73 (Supplementary Table S2). Biosynthesis of other secondary metabolites, metabolism of co-factors and vitamins, metabolism of terpenoids and polyketides, and xenobiotics biodegradation and metabolism pathways were enriched too (Supplementary Table S2). These results suggested that the difference in EC induction capability between the two inbred lines was very complex and involved various metabolic pathways. To reveal how the significantly metabolites were regulated, proteomic analysis was then conducted.

### Overview of the Proteomic Data Sets

**Figure 2:**
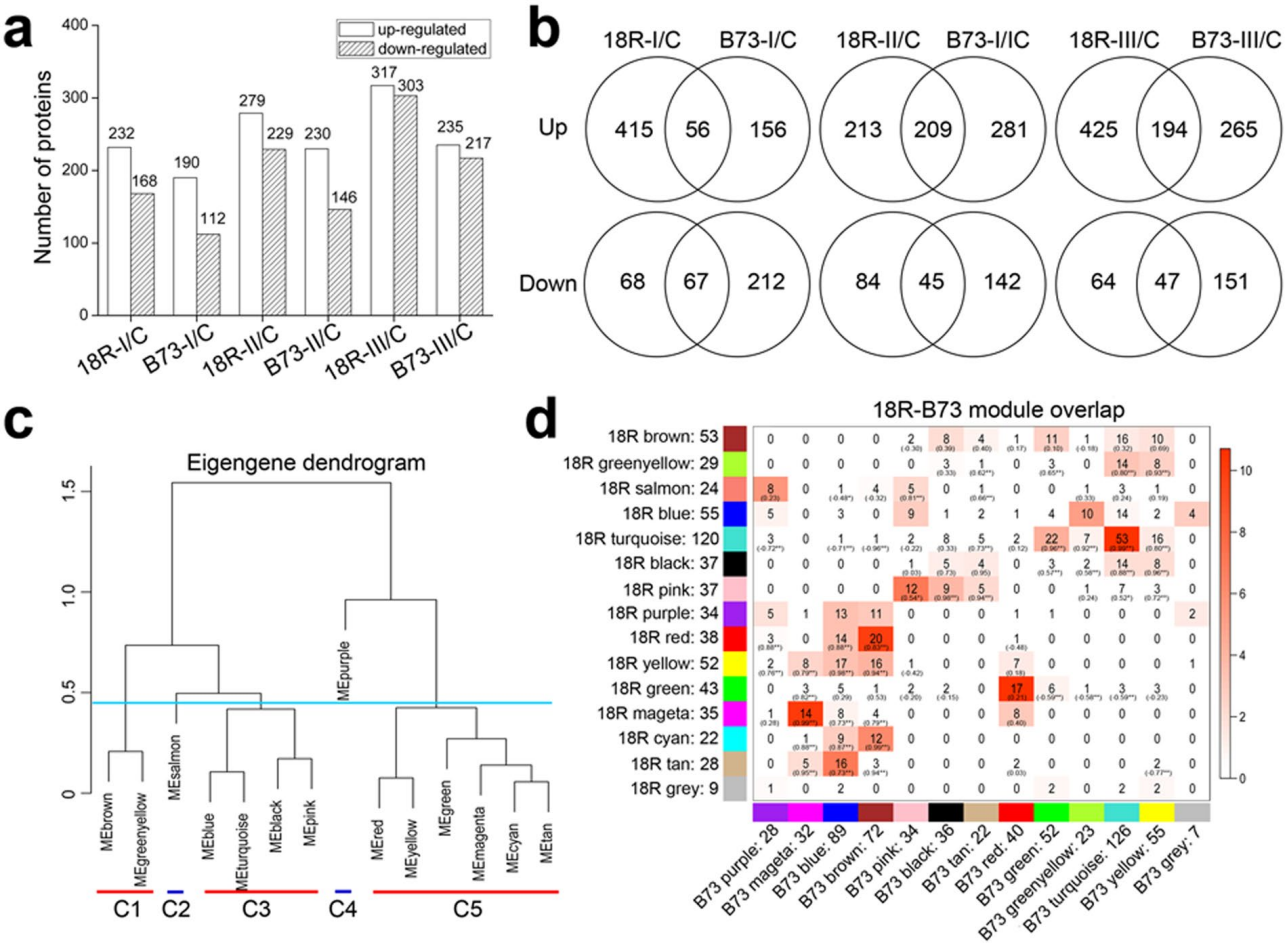
Overview of the maize proteomic response to induction medium and co-expression network analysis. (**a**) Numbers of differentially up- or down-regulated proteins (fold change >1.5, *P* < 0.05) at each stage in 18R and B73. (**b**) Venn diagram of significantly up- and down-regulated proteins in 18R and B73 at each stage. (**c**) Eigengene dendrogram of modules based on the eigengene expression level of each module in 18R. The turquoise line points to the re-cluster dividing line. MEbrown and MEgreenyellow were re-clustered to cluster 1 (C1), as well as clustering C2 (MEsalmon), C3 (MEblue, MEturquoise, MEblack and MEpink), C4 (purple) and C5 (MEred, MEyellow, MEgreen, MEmagenta, MEcyan and MEtan). (**d**) Correspondence of 18R modules and B73 modules. Numbers in the table indicate protein counts at the intersection of the corresponding modules. Numbers in brackets represent the correlation coefficient of the module eigengene in the corresponding modules. *P < 0.05; **P < 0.01. Coloring of the table represents the significance of the overlap of the two modules [log (*P*), Fisher’s exact test].

Total proteins of CK, stage I, II and III of 18R and B73 were submitted to iTRAQ-based quantitative proteome analysis. A total of 5,797, 6,232 and 6,216 proteins were identified in replicates 1, 2 and 3, respectively. In this study, the 4,816 proteins identified in all 3 replicates were retained for further analysis (Supplementary Table S3). Relative to CK, the proteins with fold changes of >1.5 (or <0.67) and *P* values < 0.05 in any sample or in any line were classified as significantly changed proteins. In total, we identified 616 significantly changed proteins (Supplementary Table S4). In 18R, 232, 279 and 317 proteins were up-regulated in stage I, II, and III, respectively. In contrast, 168, 229 and 303 proteins were significantly down-regulated in stage I, II and III, respectively (Fig. 2a). In B73, however, 190, 230 and 235 proteins were up-regulated, and 112, 146 and 217 proteins were significantly down-regulated in stage I, II and III, respectively (Fig. 2a). At each stage, more proteins were either up-regulated or down-regulated in 18R than in B73. As shown in Fig. 2b, only 75 (22%), 128 (34%) and 118 (27%) proteins were significantly up-regulated at stage I, II and III in both 18R and B73, respectively. Similarly, only 48 (21%), 89 (31%) and 124 (31%) proteins were significantly down-regulated in three stages in both lines, respectively. These results suggested that 18R and B73 responded to induction medium by expressing different proteins, which could be responsible for the different EC induction capability.

### Co-expression Networks of Significantly Changed Proteins

To study the expression pattern, 616 significantly changed proteins were subjected to co-expression networks analysis using the WGCNA R package^14^. This unsupervised and unbiased analysis identified 14 and 12 distinct co-expression modules in 18R and B73, respectively (Fig. 2d, Supplementary Fig. S2a, and Supplementary Table S4). We then performed a modules-preservation test using repeated random sampling and modules recalculation. All the *Zsummary* scores of the modules of the two inbred lines were larger than 6 (Supplementary Table S5). According to previous study^15^, modules with *Zsummary* scores >2 were moderately preserved, which indicated that all of the modules in our study were conserved. The module preservation of 18R modules in the B73 data was calculated, which can also be regarded as a differential network analysis. The salmon, blue, green and purple modules of 18R with Zsummary scores of <2 cannot be recalculated in B73 data (Supplementary Fig. S2b). Accordingly, 150 proteins in these 4 modules were selected as differentially expressed proteins (DEPs) between 18R and B73.

We also compared the B73 modules with the 18R modules and calculated the correlation of eigengenes value (the first principal component of the protein profiles) for each module between 18R and B73 (Fig. 2d). As expected, the proteins in most of the 18R modules overlapped with several different B73 modules, and most of the correlations resulted in very high values. Finally, 93 proteins with correlation of <0.4 were selected as DEPs, too. In total, 243 DEPs (Supplementary Table S4: red marked proteins) between 18R and B73 were chosen for further analysis.

### EC Formation-Related DEP Annotation

Based on the eigengene dendrogram (Fig. 2c), the MEbrown and MEgreenyellow modules had very similar expression profiles, indicating that their genes were highly co-expressed; thus, these modules were merged and renamed as cluster 1 (C1). Similarly, MEblue, MEturquoise, MEblack and MEpink were merged and renamed as cluster 3 (C3), whereas MEred, MEyellow, MEgreen, MEmagenta and MEtan were merged and renamed as cluster 5 (C5). MEsalmon and MEpurple were renamed as cluster 2 (C2) and cluster 4 (C4), respectively.

**Figure 3:**
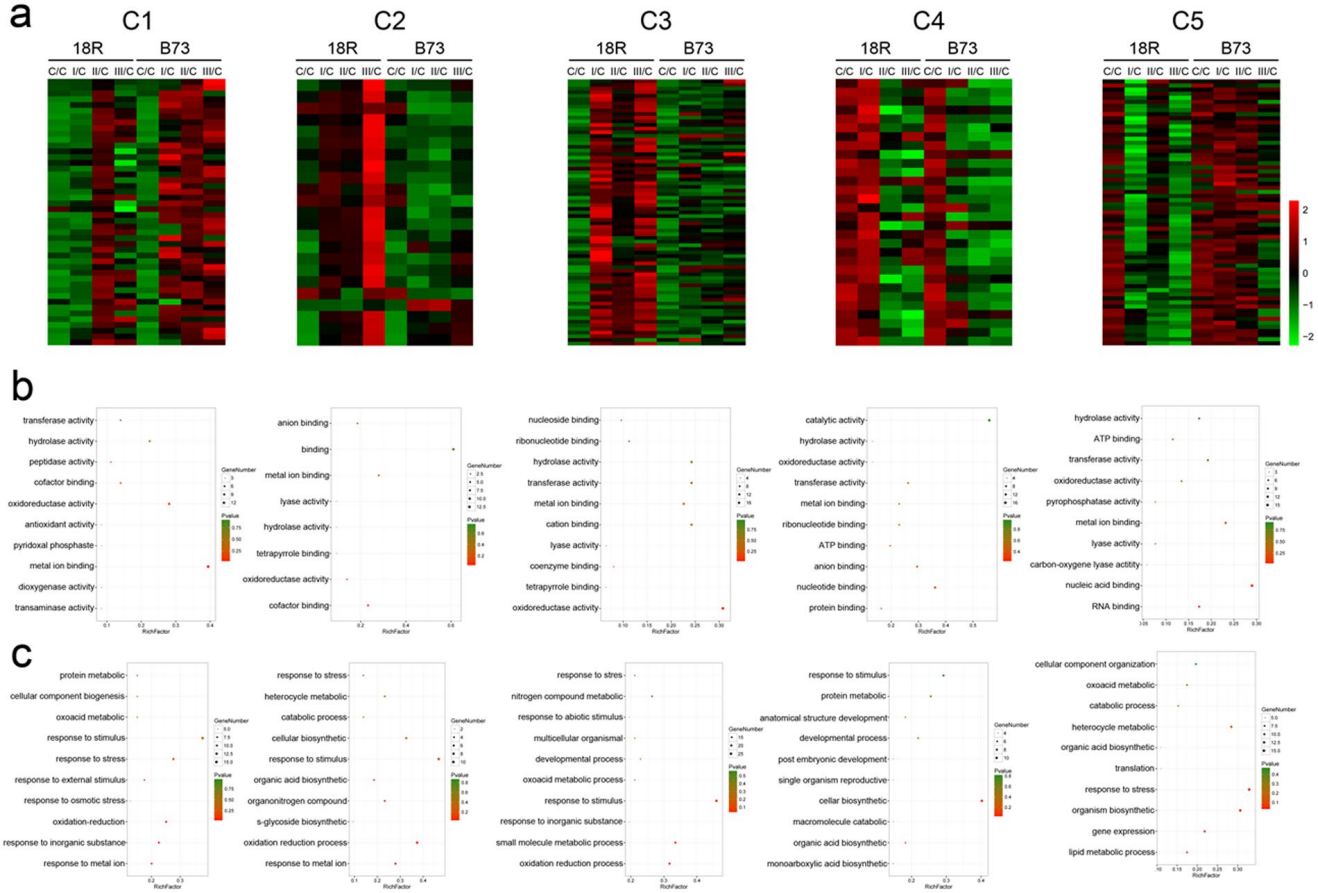
Cluster information. (**a**) Heatmap of the differentially expressed proteins (DEPs) between 18R and B73 in each cluster. (**b**) The top ten GO terms of molecular function of each cluster. (**c**) The top ten GO terms of biological process terms of each cluster.

As shown in Fig. 3a, the C1 DEPs in 18R were substantially up-regulated in stage II, and there were no significant changes in stages I and III. However, the DEPs in B73 increased at all 3 stages. These DEPs likely played an important role in the initial EC formation and were not required for stage I or III. In C2, DEPs were up-regulated at 3 stages in 18R and especially increased at stage III, whereas no significantly changes were noted in B73. The DEPs in C3 were sharply up-regulated during all stages in 18R, but not even down-regulated in B73. On the contrary, the DEPs in C5 decreased at stage I and retained a lower level at stages II and III in 18R, but did not significantly change in B73, which suggested that these DEPs are rapid and continuous response factors. The C4 DEPs did not change at stage I, but were extremely reduced at stages II and III in 18R. Still, they were sharply down-regulated at all stages in B73, which suggested that they were needed for embryo enlargement in 18R.

To understand the role of differentially expressed genes in EC, the 243 DEPs were submitted to Gene Ontology (GO) enrichment analysis. The top ten GO terms of each cluster are shown in Fig. 3b and c. These molecular functions and biological process GO terms include metal ion binding, oxidoreductase activity, protein binding, RNA binding, lyase activity, hydrolase activity, metabolic processes, response to stimulus and metal ions, oxidation-reduction processes, and organonitrogen compound metabolics (Supplementary Table S6).

Interestingly, a total of 69 DEPs in C1, C2, C3, C4 and C5 were enriched for metal ion binding or corresponding functions (*P* < 0.05, Table 1). Among them, four dehydrogenase family proteins were induced in all of the stages (Table 1). Likewise, three cytochrome family proteins were up-regulated, while two were down-regulated (Table 1). Six peroxidase super family proteins in C1, C2 or C3 (Table 1) were up-regulated in the process of EC formation. These DEPs may function by activating oxygen scavenging. In addition, three DEPs were glutathione S-transferase-like proteins, which are widely known to respond to stimuli or detoxification in scavenging of toxic organic hydroperoxides to protect plants from ROS damage^16^. Similarly, genes involved in energy metabolism were induced at all 3 stages (Table 1 and Fig. 3). Clearly, EC formation is a complex process that requires sufficient energy for fast growth and cell division^17^ so that energy metabolism was already identified as important for maize and Arabidopsis callus induction^10^.

**Table 1:** Metal ion response or binding DEPs.

Protein id	Description	Log_2_FC-18R			Log_2_FC-B73		
		I/C	II/C	III/C	I/C	II/C	III/C
GRMZM2G105005_P01	Glutathione S-transferase	1.23	0.94	1.86	−0.34	−0.43	0.81
GRMZM2G434541_P01	Glutathione S-transferase	2.39	0.71	0.98	0.54	0.22	0.51
GRMZM2G475059_P01	Glutathione S-transferase	1.93	1.83	1.60	1.70	0.95	0.93
GRMZM2G003883_P01	Pyruvate kinase family	0.92	1.01	1.31	0.32	0.67	1.00
GRMZM2G008714_P01	Pyruvate kinase family protein	−0.15	−0.42	0.26	−2.40	−2.38	−2.09
GRMZM2G097457_P01	pyruvate	−0.78	−1.32	−1.68	0.60	−0.75	−1.57
GRMZM2G010044_P01	isopropyl malate isomerase	0.08	0.03	0.60	−0.18	−0.24	−0.27
GRMZM2G031107_P01	glucose-6-phosphate dehydrogenase 6	1.17	0.99	2.22	−0.32	−0.30	0.23
GRMZM2G063851_P01	cts1 - citrate synthase1	0.91	0.58	1.05	0.73	0.64	0.94
GRMZM2G138468_P01	amya3 - alpha amylase3	0.99	1.00	2.28	−0.28	0.05	1.19
GRMZM2G157018_P01	ATP synthase D chain mitochondrial	−0.33	0.50	−0.02	0.50	0.74	0.80
GRMZM2G172369_P03	Glycosyl hydrolase family 38	0.13	0.67	0.32	0.54	0.66	0.38
GRMZM2G100146_P01	Histone deacetylase HDT2	−1.12	−0.38	−0.69	−0.22	−0.55	−0.78
GRMZM2G159032_P01	Histone deacetylase HDT3	−1.01	−0.30	−0.85	−0.36	−0.40	−0.65
GRMZM2G450206_P01	RNA recognition motif	−0.32	−0.87	−1.13	−0.11	−0.30	−0.18
GRMZM2G026855_P04	Zinc-binding dehydrogenase	0.45	−0.12	0.38	−0.56	−0.72	−0.63
GRMZM2G046070_P01	cinnamyl alcohol dehydrogenase1	−0.20	−1.10	−0.91	−0.93	−1.21	−1.22
GRMZM2G058584_P01	histidinol dehydrogenase	−0.06	−0.34	0.30	−0.83	−0.73	−0.89
GRMZM2G098346_P01	adh2 - alcohol dehydrogenase2	−0.18	1.15	0.79	−0.06	1.05	1.64
GRMZM2G139512_P02	alcohol dehydrogenase 1	0.43	0.20	0.90	−0.26	0.03	0.15
GRMZM2G147191_P02	ARP protein (REF)	0.90	0.63	1.22	0.43	0.50	0.69
GRMZM2G024234_P01	Peroxidase superfamily protein	0.63	0.37	2.48	−0.04	−0.24	0.11
GRMZM2G040638_P01	pox2 - guaiacol peroxidase2	0.27	1.80	1.30	0.50	1.77	2.62
GRMZM2G047456_P02	Peroxidase superfamily protein	1.00	1.00	1.62	0.53	0.60	0.77
GRMZM2G104394_P01	pox1 - guaiacol peroxidase1	0.29	0.69	0.42	0.29	0.63	1.02
GRMZM2G129761_P01	1-cysteine peroxiredoxin 1	−1.39	−1.27	−2.30	−0.32	−0.26	−1.78
GRMZM2G138450_P01	Peroxidase superfamily protein	1.59	1.38	2.82	−0.54	−0.80	−0.49
GRMZM2G102760_P01	lox5 - lipoxygenase5	0.38	0.63	1.04	0.23	0.57	1.41
GRMZM2G015419_P02	lox10 - lipoxygenase10	0.66	1.12	0.44	1.10	1.62	2.73
GRMZM2G002178_P01	aos2 - allene oxide synthesis2	−0.58	−0.17	−0.98	0.23	0.07	−0.10
GRMZM2G014395_P01	CYP72A14	1.07	0.95	2.52	0.15	0.13	0.33
GRMZM2G075244_P01	CYP709B2	0.12	−1.21	−0.92	−0.77	−1.30	−1.27
GRMZM2G134738_P01	Rubredoxin-like superfamily protein	0.20	0.75	0.23	0.87	0.33	0.41
GRMZM2G167549_P05	bx3 - benzoxazinone synthesis3	0.81	0.77	0.72	−0.03	0.42	0.68
GRMZM2G024104_P01	gln2 - glutamine synthetase2	0.33	0.59	1.07	0.66	0.30	0.44
GRMZM2G088627_P01	Peptidase M20/M25/M40 family protein	−0.11	0.42	0.09	0.67	0.68	0.86
GRMZM2G080828_P01	ornithine-delta-aminotransferase	0.01	0.34	0.15	0.69	0.67	0.45
GRMZM5G826838_P01	glutamate decarboxylase	1.38	1.06	1.87	−0.21	−0.43	0.01
GRMZM2G124353_P01	alanine:glyoxylate aminotransferase 2	0.11	0.61	0.02	1.42	0.76	0.03
GRMZM2G047732_P01	40 S ribosomal protein S27a	−0.65	−0.18	−0.95	−0.20	−0.10	−0.35
GRMZM2G081310_P01	calcium dependent protein kinase7	0.68	−0.81	−0.49	−0.81	−0.80	−0.91
GRMZM2G048324_P01	nrx1 - nucleoredoxin1	0.27	1.27	1.28	0.91	1.14	1.54
GRMZM2G135132_P01	adenosine kinase	1.24	1.01	1.98	−0.41	−0.66	0.87
GRMZM2G134797_P02	ndk1 - nucleotide diphosphate kinase1	0.79	0.92	1.74	1.07	0.56	0.74
AC148152.3_FGP005	2-oxoglutarate (2OG)	0.10	0.92	0.19	0.17	1.22	3.46
GRMZM2G078500_P01	aromatic ring-opening dioxygenase	2.00	1.21	1.59	−0.10	−0.17	0.21
GRMZM2G054559_P01	pld1 - phospholipase D1	0.89	0.66	1.24	−0.02	0.17	0.52
GRMZM2G054905_P01	Insulinase	−0.21	0.81	−0.17	0.36	0.75	1.13
GRMZM2G325575_P01	fer1 - ferritin1	−0.36	0.42	0.90	−0.83	0.10	0.78
AC232238.2_FGP005	ferredoxin 3	1.02	1.10	1.99	0.21	0.45	1.46
GRMZM2G087259_P01	alpha carbonic anhydrase 7	1.15	0.33	1.12	0.40	0.52	0.58
GRMZM2G113332_P01	copper chaperone	−0.43	0.50	−0.09	1.34	1.01	0.86
AC191050.3_FGP003	cupin family protein	−0.17	1.74	−0.55	2.34	2.41	−0.18
GRMZM2G106424_P01	pza03081 -	0.80	−0.55	0.57	−2.02	−1.52	−1.54
GRMZM2G008290_P01	Heavy metal transport	−0.29	−0.54	−0.57	−0.12	−0.25	−0.58
GRMZM2G127609_P01	chaperonin 20	−0.77	−0.38	−0.86	0.25	−0.15	−0.57
GRMZM2G081571_P04	Molybdenum cofactor sulfurase	−0.10	0.79	0.90	0.53	0.69	0.79
GRMZM2G153541_P06	elfa9 - elongation factor 1-alpha9	−0.68	−0.31	−0.31	0.05	0.39	0.18
GRMZM2G070863_P01	stress-inducible protein putative	0.34	0.91	0.01	1.11	1.05	0.91
GRMZM2G162388_P01	cis-trans isomerase	−1.83	−0.30	−1.97	−0.06	0.25	1.16
GRMZM2G165747_P01	5-methyltetrahydropteroyltriglutamate	0.04	0.80	0.74	0.71	0.42	0.54
GRMZM2G176707_P01	nucleosome assembly protein 1;2	0.12	1.10	0.02	1.14	1.15	0.74
GRMZM2G332522_P01	pza01810 -	1.58	2.02	3.64	1.72	1.62	2.30
GRMZM2G035620_P01	alpha/beta-Hydrolases	0.21	−0.37	0.71	−0.88	−1.08	−0.49
GRMZM2G125196_P01	Zinc-binding dehydrogenase	3.49	2.55	2.54	3.19	1.80	1.64
GRMZM2G408768_P01	general regulatory factor 2	0.89	−0.07	−0.08	−0.06	−0.15	−0.40
GRMZM2G066460_P01	60 S acidic ribosomal protein P0	0.50	−0.25	0.49	−1.58	−2.08	−2.15
GRMZM2G075255_P01	Fatty acid hydroxylase superfamil	−0.61	−0.56	−1.10	−0.66	−0.95	−0.60
GRMZM2G031825_P01	Metallo-hydrolase/oxidoreductase	−0.77	0.08	−0.03	−0.12	0.00	−0.11

By GO analysis, 30 DEPs were classified into cell differentiation, division, communication and programmed cell death (Table 2). Although their *P* values were larger than 0.05, we selected them for further analysis because the process of EC induction does involve cell dedifferentiation, division and re-differentiation. Among them, 4 reduced and 4 increased DEPs were assigned to cell-cell junction or cell communication (Table 2). Meanwhile, 3 increased and 2 reduced DEPs were classified as cell differentiation, and 3 down-regulated DEPs as players in cell division (Table 2). Moreover, GRMZM2G408768_P01 was annotated as a cell cycle regulator, which was up-regulated at stage I in 18R but immediately reduced to normal levels at following stages (Table 2). However, the level of this protein did not increase at stage I and even decreased at stages II and III in B73.

**Table 2:** Cell division, differentiation and dedifferentiation process related DEPs.

Protein id	GO	Log_2_FC-18R			Log_2_FC-B73		
		I/C	II/C	III/C	I/C	II/C	III/C
AC208897.3_FGP004	cell-cell junction	2.49	1.66	2.57	0.27	0.65	0.88
GRMZM2G074102_P01	cell-cell junction	0.77	−0.05	0.22	−0.04	−0.03	−0.08
GRMZM2G010991_P01	cell-cell junction	0.12	−0.78	−0.40	−0.86	−0.88	−1.09
GRMZM2G377215_P01	cell-cell junction	−0.97	−0.48	−0.96	−0.31	−0.45	−0.41
GRMZM2G108149_P01	cell-cell junction	−0.47	−0.70	−0.90	−0.02	0.01	−0.25
GRMZM2G053669_P01	cell communication	0.22	1.18	0.21	2.51	1.23	0.21
GRMZM2G468756_P01	cell communication	0.79	0.28	0.34	0.65	0.73	0.69
GRMZM2G133926_P01	cell communication	−0.84	−0.55	−0.73	−0.17	−0.37	−0.86
GRMZM2G048324_P01	cell differentiation	0.27	1.27	1.28	0.91	1.14	1.54
GRMZM5G826838_P01	cell differentiation	1.38	1.06	1.87	−0.21	−0.43	0.01
GRMZM2G099666_P01	cell differentiation	1.83	1.54	1.56	0.84	1.23	1.28
GRMZM2G124239_P01	cell differentiation	0.30	−0.32	−0.10	−0.76	−0.65	−0.79
GRMZM2G006474_P01	cell differentiation, cell division	−0.70	−0.10	−0.07	0.32	0.69	0.39
GRMZM2G143627_P01	cell division	−0.02	−0.64	−0.56	−0.84	−0.72	−0.85
GRMZM2G333916_P01	cell division	−0.26	−0.74	−1.02	−0.67	−0.82	−1.14
GRMZM2G408768_P01	cell cycle	0.89	−0.07	−0.08	−0.06	−0.15	−0.40
GRMZM2G393272_P02	meristem development	1.13	0.36	0.73	0.40	0.56	0.47
GRMZM2G109284_P01	meristem initiation	0.86	0.55	0.47	−0.24	0.15	−0.40
GRMZM2G129189_P01	somatic embryogenesis	1.35	1.16	1.99	0.40	0.13	0.86
GRMZM2G082199_P01	regulation of cell death	0.19	0.27	0.82	−0.43	−0.22	−0.01
GRMZM2G113332_P01	regulation of cell death	−0.43	0.50	−0.09	1.34	1.01	0.86
GRMZM2G022931_P01	plant-type cell wall	0.12	1.77	2.00	0.85	1.24	1.80
GRMZM2G172369_P03	plant-type cell wall	0.13	0.67	0.32	0.54	0.66	0.38
GRMZM2G019411_P01	plant-type cell wall	0.20	0.10	0.74	−0.26	−0.45	0.01
GRMZM2G121514_P01	plant-type cell wall	1.00	0.32	0.68	0.32	0.20	0.16
GRMZM2G066274_P01	plant-type cell wall	−0.66	−0.54	−1.04	0.00	−0.09	−0.40
GRMZM2G012224_P01	plant-type cell wall	−0.92	−0.58	−1.49	−0.26	−0.47	−1.01
GRMZM2G157263_P01	plant-type cell wall	−0.42	0.52	−0.34	0.63	0.97	0.89
AC234156.1_FGP005	plant-type cell wall	−0.92	−0.02	−0.57	0.12	−0.13	−0.22
GRMZM2G413006_P01	plant-type cell wall	−0.38	0.32	−0.33	0.28	0.52	0.68

In addition, the RNI-like protein superfamily (GRMZM2G393272_P02), including the SART-1 (GRMZM2G109284_P01) and Endochitinase PR4 proteins (GRMZM2G129189_P01) were assigned to the biological processes of meristem development, meristem initiation and somatic embryogenesis, respectively (Table 2). These 3 DEPs of 18R were dramatically increased during all stages; however, they did not significantly change in B73, or the fold-change in B73 was smaller than in 18R (Table 2). Moreover, 4 up-regulated and 5 down-regulated DEPs in 18R (Table 2) were predicted as plant-type cell wall related genes, which were required during cell division.

KOBAS (http://kobas.cbi.pku.edu.cn/home.do) was used to perform pathway analysis. Similar to GO analysis, energy metabolism-related pathways were identified (Table 3), which suggested that EC induction required a lot of energy. As expected, amino acid synthesis and metabolism pathways were also enriched. In addition, hormone-related pathways were identified, such as plant hormone signal transduction, brassinosteroid biosynthesis, alpha-linolenic acid metabolism (which participates in jasmonic acid biosynthesis and zeatin biosynthesis), cysteine and methionine metabolism (which participates in ethylene biosynthesis) and tryptophan metabolism (which is involved in auxin biosynthesis) (Table 3).

**Table 3:** Pathways annotated from all DEPs.

Pathway	Pathway ID	P value	Q value
alpha-Linolenic acid metabolism	ko00592	0.00	0.00
Other glycan degradation	ko00511	0.00	0.03
Cysteine and methionine metabolism	ko00270	0.00	0.03
Phenylpropanoid biosynthesis	ko00940	0.01	0.27
Fatty acid degradation	ko00071	0.02	0.28
Biosynthesis of amino acids	ko01230	0.02	0.28
Fatty acid metabolism	ko01212	0.02	0.28
Brassinosteroid biosynthesis	ko00905	0.02	0.28
Monoterpenoid biosynthesis	ko00902	0.02	0.28
Glutathione metabolism	ko00480	0.04	0.39
Selenocompound metabolism	ko00450	0.06	0.50
2-Oxocarboxylic acid metabolism	ko01210	0.06	0.50
Linoleic acid metabolism	ko00591	0.07	0.50
Valine, leucine and isoleucine biosynthesis	ko00290	0.08	0.55
Fatty acid biosynthesis	ko00061	0.09	0.55
Zeatin biosynthesis	ko00908	0.20	0.84
Carotenoid biosynthesis	ko00906	0.30	0.84
Phenylalanine metabolism	ko00360	0.51	0.85
Phenylalanine, tyrosine and tryptophan biosynthesis	ko00400	0.65	0.92
Tryptophan metabolism	ko00380	0.70	0.93
Plant hormone signal transduction	ko04075	0.78	0.94

### Plant Hormone-Related DEPs Induced by Auxin-containing Medium

Both the GO and KEGG pathway annotation suggested that hormone signaling transduction-related pathways were activated. To identify whether the proteins are regulated by hormones, the Hormonometer program was used to compare the level of protein fold changes with indexed data sets treated by hormones^18^ (http://hormonometer.weizmann.ac.il/hormonometer). In Arabidopsis (*Arabidopsis thaliana*), the gene expression profiles treated by methyl jasmonate, ethylene, abscisic acid, indole-3-acetic acid, cytokinin (zeatin), brassinosteroid, GA3 and salicylic acid have already been completed. To carry out this analysis, only the proteins having its Arabidopsis homologs and containing probe set identifiers (required by Hormonometer) were kept. A total of 2,750 Arabidopsis homologs of maize proteins (Supplementary Table S7) were submitted to Hormonometer analysis.

**Figure 4:**
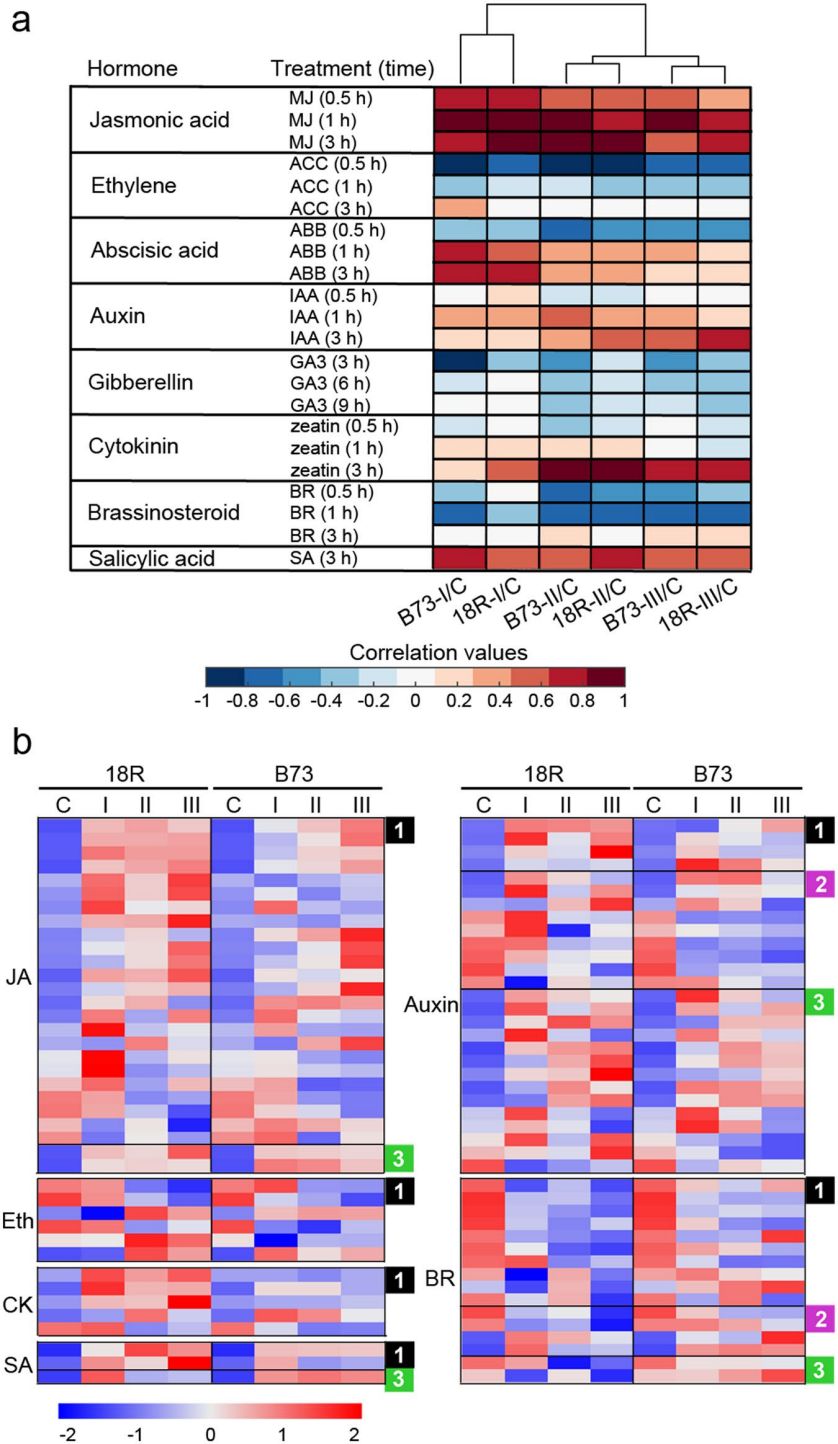
Hormone-regulated protein analysis based on the proteomic data. (**a**) The hormone response genes analyzed by Hormonometer program^18^ based on the Arabidopsis homolog. Red color represents a positive correlation between the maize protein response to induction medium and to different hormones, while blue color represents a negative correlation. (**b**) Relative expression patterns of hormone-related proteins. Each hormone-related proteins are divided into three functional categories^19^: 1, synthesis-degradation; 2, signal transduction; and 3, induced- or regulated- or responsive- activated.

As expected, during EC formation changes of protein expression were associated with hormone signaling (Fig. 4a). Based on the dendrogram, protein expression changes at stage I were distinct from those observed at stages II and III, and changes in 18R and B73 were very comparable at each stage (Fig. 4). Moreover, there was a strong positive correlation between the proteins induced by hormone-containing medium and the Arabidopsis homologous genes induced for 0.5, 1 and 3 h with jasmonic acid and for 3 h with salicylic acid. Similarly, a positive correlation was observed between the expression changes of maize protein induced by hormone-containing medium and the Arabidopsis genes induced for 1 and 3 h by auxin. Notably, at stage II and III, the correlation of 18R at 3 h auxin treatment was enhanced compared to that of B73. A similar positive correlation was identified after cytokinin treatment for 3 h, and the correlation in 18R was enhanced compared to that of B73 for stage I. On the contrary, there was a significant negative correlation between maize proteins and Arabidopsis genes treated with ethylene for 0.5 h and 1 h, brassinosteroid for 0.5 h and 1 h, GA3 for 3 h, 6 h and 9 h. Moreover, a positive correlation, especially at stage I, was observed for maize proteins induced by induction medium and Arabidopsis genes treated with ABA for 1 and 3 h. However, a strong negative correlation was identified after ABA treatment for 0.5 h at all 3 stages in both 18R and B73.

During maize leaf development, 431 expressed genes are identified to be relevant to hormone synthesis, transduction and responding^19^. In the present study, 83 expressed proteins were involved in hormone related pathways (Fig. 4b). During callus formation, 18R and B73 show different abilities in synthesis, transduction or responding of Jasmonic acid (JA), ethylene (Eth), cytokinin (CK), salicylic acid (SA), auxin, and brassinosteroid (BR).

### Different Hormone Biosynthesis Patterns in 18R and B73

**Figure 5:**
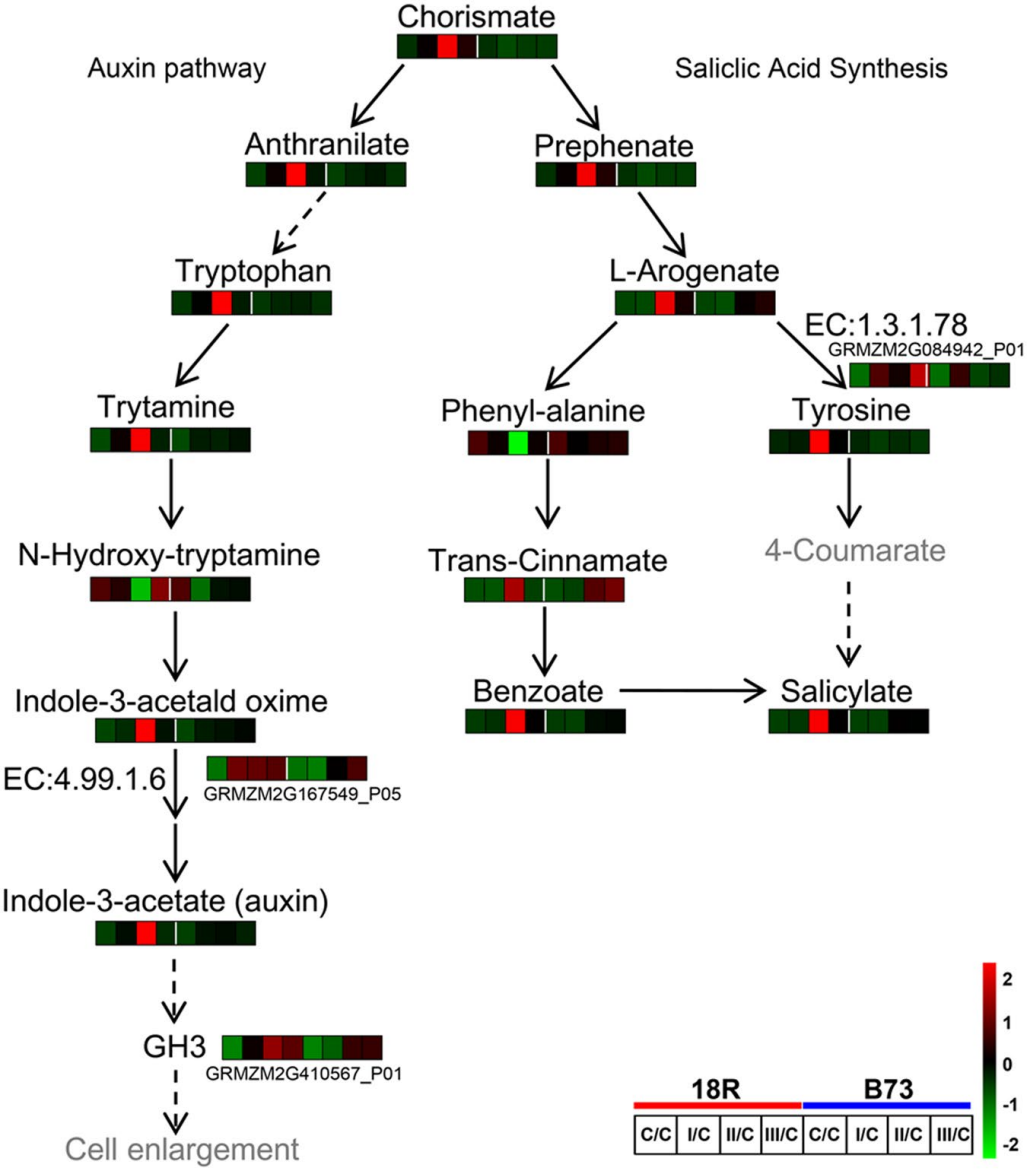
Auxin- and salicylic acid-related pathway changes induced by induction medium. The heatmap represents the metabolite and DEP abundance at different stages of 18R and B73. The dashed arrow represents multiple reaction steps.

The phenylalanine, tyrosine and tryptophan biosynthesis pathways are involved in the synthesis of chorismate, which is necessary for auxin and salicylate synthesis in plants (Fig. 5). Compared to the control, chorismate levels were significantly increased in 18R at all 3 stages, but not in B73 (Fig. 5). Furthermore, the levels of anthranilate, tryptophan, tryptamine, N-hydroxyl-tryptamine, indole-3-acetaldehyde oxime and indoleacetaldoxime dehydratase (EC: 4.99.1.6, GRMZM2G167549_P05) were all increased at least at one stage in 18R, but not in B73. These changes led to the increase of indole-3-acetic acid (auxin) in 18R, but no changes in B73. Moreover, in the auxin transduction pathway, a GH3 gene family protein, GRMZM2G410567_P01, was significantly up-regulated at 3 stages in both lines, but changed more in 18R than in B73. In the salicylic acid synthesis pathway (Fig. 5), prephenate, L-arogenate, trans-cinnamate, benzoate and tyrosine and arogenate dehydrogenase (EC: 1.3.1.78, GRMZM2G084942_P01) were all up-regulated at least in one stage in 18R, but with no significant changes in B73, which led to a significant increase of salicylate in 18R at stages II and III, but not in B73.

**Figure 6:**
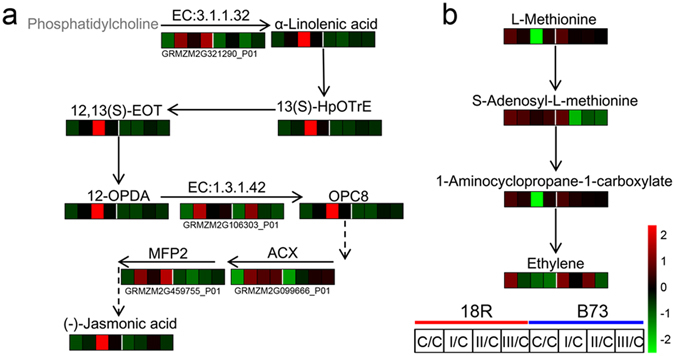
Effects of medium induction on the proteins and metabolites of the jasmonic acid (**a**) and ethylene (**b**) synthesis pathways. The heatmap represents the metabolite and DEP abundance at different stages of 18R and B73. The gray metabolite was not detected or did not significantly change.

Similarly, the activation of the jasmonic acid pathway was identified under metabolite pathways (Supplementary Table S2) and hormone-regulated gene expression analysis (Fig. 4). Therefore, we further expanded a detailed analysis of the jasmonic acid synthesis pathway. As shown in Fig. 6a, HRAS-like suppressor 3 (EC: 3.1.1.32, GRMZM2G321290_P01), as well as α-linolenic acid, 13(S)-HpOTrE, 12,13(S)-EOT, 12-OPDA, 12-oxophytodienoic acid reductase (EC: 1.3.1.42, GRMZM2G106303_P01), OPC8, acyl-CoA oxidase (ACX, GRMZM2G099666_P01, EC: 1.3.3.6), and enoyl-CoA hydratase/3-hydroxyacyl-CoA dehydrogenase (MFP2, GRMZM2G459755_P01, EC: 4.2.1.17) increased at 3 stages and peaked at stage II in 18R, but there was no significant change in B73, which resulted in an increase of the production of (-)-jasmonic acid in 18R, but not in B73.

**Figure 7:**
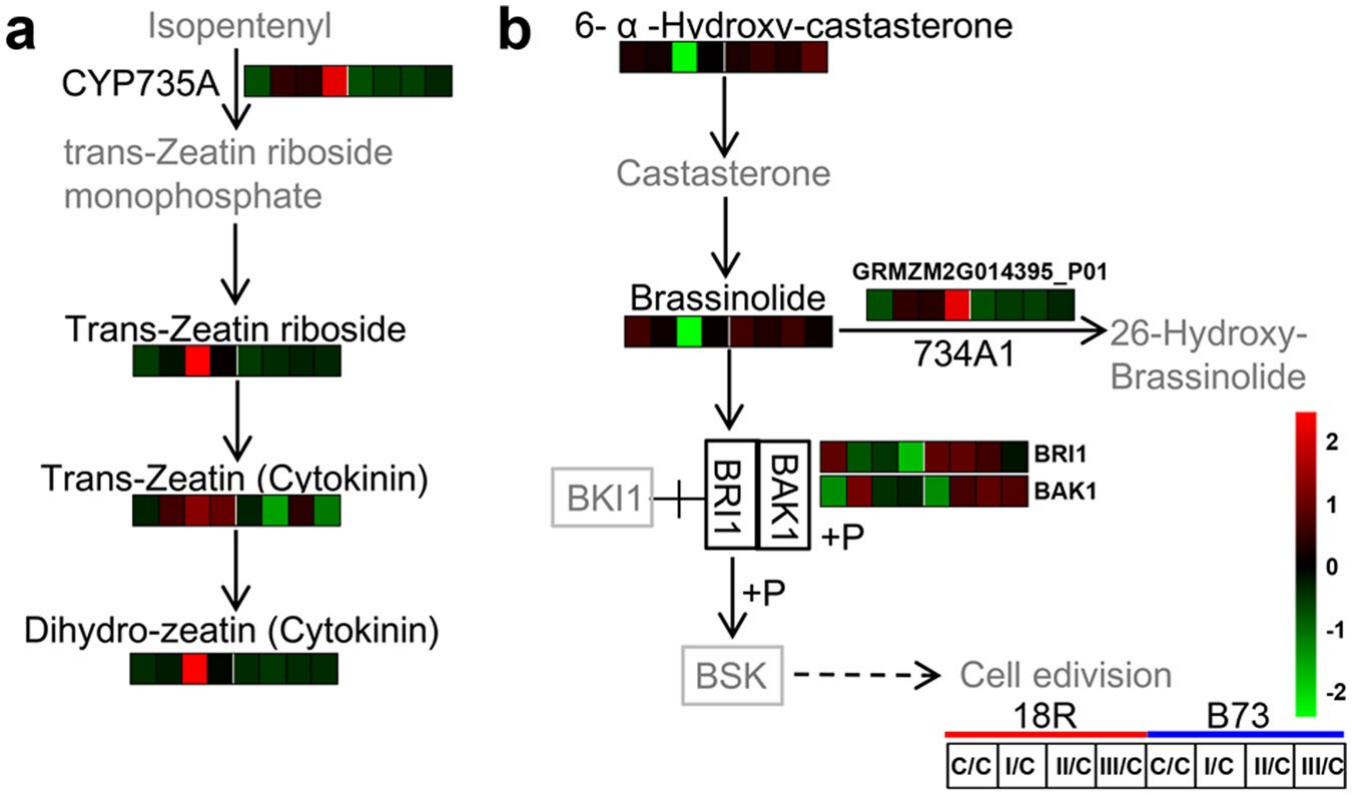
Effects of medium induction on the cytokinin synthesis pathway and the brassinolide synthesis and transduction pathways. The heatmap represents the metabolite and DEP abundance at different stages of 18R and B73. The gray metabolite was not detected or did not significantly change.

Different concentrations of cytokinin are usually added for *in vitro* callus induction. Hormone-regulated protein analysis showed that several proteins were regulated by cytokinin (Fig. 4). Therefore, we also focused on the cytokinin synthesis pathway (Fig. 7a). As expected, cytokinin trans-hydroxylase (CYP735A, GRMZM2G014395_P01), trans-zeatin riboside, zeatin (cytokinin), and dihydrozeatin (cytokinin) were all significantly up-regulated at all stages in 18R but were unchanged in B73, which suggested that 18R and B73 have different cytokinin synthesis capabilities.

In contrast to these hormones, the brassinolide synthesis and transduction pathway were repressed (Figs 4 and 7b). The levels of 6-α-hydroxy-castasterone and brassinolide were reduced at all 3 stages and peaked at stage II in 18R, but did not change in B73. Moreover, the up-regulation of CYP734A1 (PHYB activation tagged suppressor 1, EC:1.14.-.-, GRMZM2G014395_P01) in 18R led brassinolide to be modified to 26-hydroxy-brassinolide, which further lowered its concentration, whereas there were no changes in B73. Furthermore, BRI1 (EC: 2.7.10.1, GRMZM2G066274_P01) significantly decreased in the brassinolide transduction pathway at all stages in 18R with no significantly changes in B73. However, BAK1 (GRMZM2G468756_P01, EC: 2.7.10.1) was significantly up-regulated at stage I in 18R and at all stages in B73. These results suggested that the brassinolide synthesis and transduction pathway were suppressed in 18R but not in B73.

Likewise, ethylene biosynthesis was also suppressed during EC induction. As shown in Fig. 6b, except for S-adenosyl-L-methionine, which level was more reduced in B73, the level of other metabolites including L-methionine, 1-aminocyclopropane-1-carboxylate and ethylene decreased more in 18R than in B73 during EC induction. Ethylene response factors (ERF) function in ethylene-mediated signaling transduction^20^. In Arabidopsis, AtHD2A can directly interact with tomato Pti4 transcription factor (ERF family proteins) to further inhibit the expression of downstream genes by binding to the GCC-box region of promoter^21^. Similarly, *Longan fruit* DIERF1 also can directly interact with DIHD2^22^. HD2 is a plant-specific histone deacetylase and involved in many processes, such as seed dormancy^23^, ABA and salt stress response^24^.

**Figure 8:**
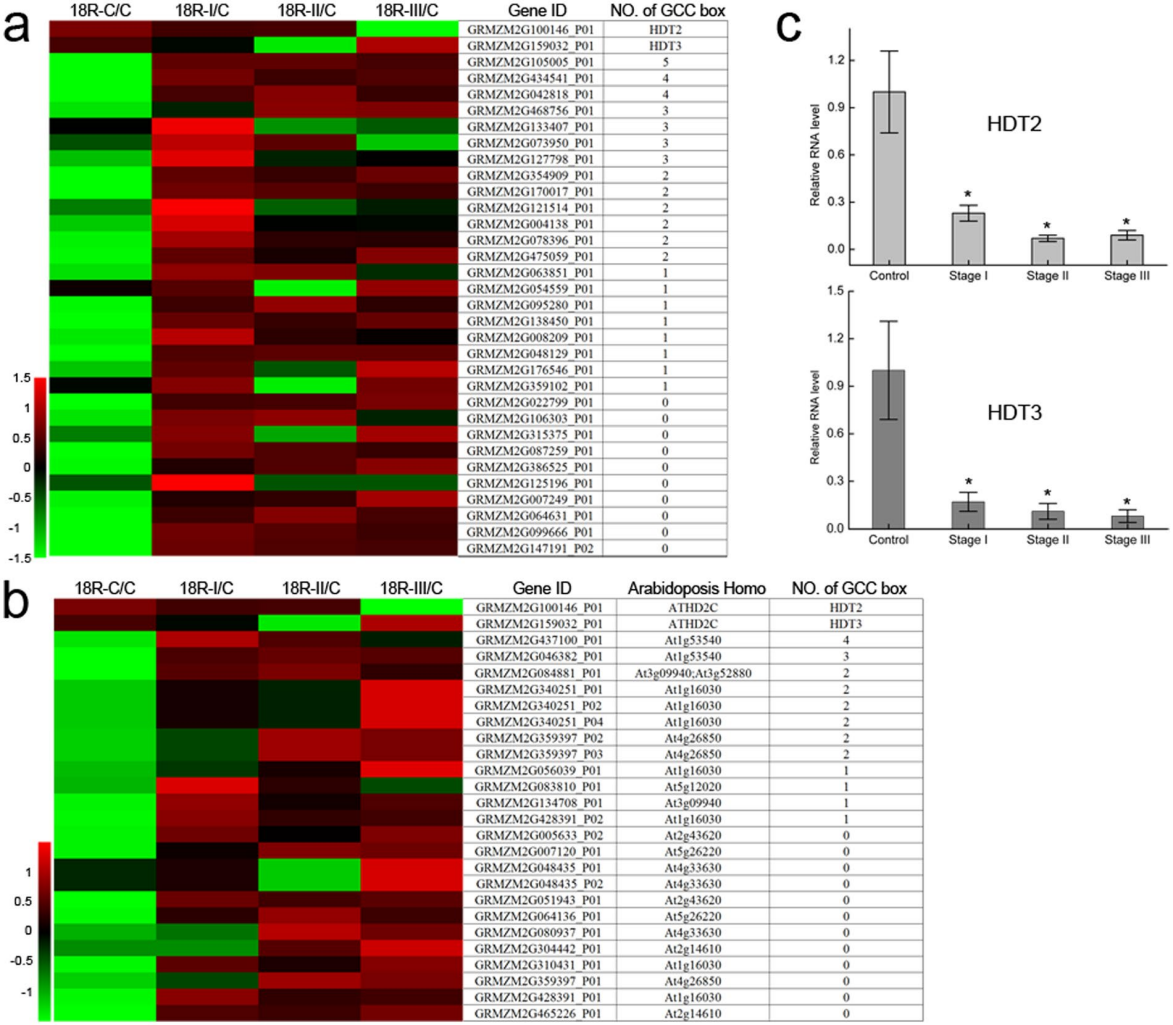
Expression pattern of genes potentially regulated by histone deacetylase. (**a**) Heatmap of proteins in the 18R blue module that show a significantly negative correlation with HDT2 (GRMZM2G100146) and HDT3 (GRMZM2G159032). No. of GCC box is the number of “GCCGCC” sequences in the promoter (3000 bp upstream of ATG). The “GCC box” was reported to be bound by ERF to regulate gene expression. ERF can directly interact with histone deacetylases to further function. (**b**) Heatmap of transcription changes of potential downstream genes of ERF and histone deacetylase at the 3 stages of 18R. (**c**) Relative expression level of *HDT2* and *HDT3* are shown as means ± SD (n = 3) during callus formation of 18R. *P < 0.05 (Student’s t test) for differences between control and each stage.

Interestingly, relative to control, *HDT2* (GRMZM2G100146_P01, homologous to *DIHD2*) and *HDT3* (GRMZM2G159032_P01, homologous to *DIHD2*) significantly decreased both in RNA level (Fig. 8c) and protein level at all stages in 18R (Fig. 8a). Thus, we hypothesize that the ERF/HDT complex decreased because of the reduction of *HDT2* or *HDT3*, which further led to the up-regulation of downstream genes. As expected, 21 DEPs in the blue module, with a highly negative correlation (<−0.7) to *HDT2* and *HDT3* at the RNA level, have at least one GCC-box in their promoter (3000 bp upstream of ATG, Fig. 8a). This suggested to us that these proteins might be directly regulated by the ERF/HDT complex. The maize homologous genes of the downstream genes of AtERF6 (homolog of DIERF1) in Arabidopsis^25, 26^ were also sharply up-regulated during EC formation (Fig. 8b), and half of them contained more than 1 GCC-box in their promoter region. The blocked ethylene synthesis and reduced ERF/HDT complex might lead to the up-regulation of downstream genes and additional regulation of EC induction.

## Discussion

To investigate the dynamic changes of proteins and metabolites during maize EC formation cultured on hormone-containing induction medium, it is necessary to consider multiple induction stages and different inbred lines with different EC induction capabilities. Most previous studies of proteomic changes focused on embryonic callus and non-embryonic callus after induction for 4 weeks or even later in only one inbred line^9, 10^. However, our metabolic and proteomic results showed that a larger range of metabolites and proteins changed significantly at early stages (Fig. 1b). We also used a genetic approach by using two maize inbreds with different EC formation capabilities and found that the two different inbred lines showed geneotype-specific responses to the induction medium. Furthermore, complementing a proteomic with a metabolomics analysis provides us with a better understanding of the maize embryonic callus induction process.

Indeed, B73 and 18R showed different cell division properties. In this study, tubulin folding cofactor A (GRMZM2G143627_P01) sharply decreased at stage I in 18R but increased at stage II in B73. Arabidopsis mutations in tubulin folding cofactor failed to form microtubules, which are essential for cell division and organized cell expansion^27^. Functional loss of these genes leads to impaired cell division with normal cell growth, which further causes formation of grossly enlarged cells. So, the sharply reduced expression of GRMZM2G143627_P01 at stage I might directly promote embryo elongation while also maintaining cell division activity.

Similarly, the abilities of meristem development, meristem initiation and somatic embryogenesis differed between 18R and B73. In Arabidopsis, despite the functional loss of *ATMAX1* and *ATMAX2*, the first axillary shoots remain active during shoot formation^28^, which suggests that they repress axillary meristem differentiation. Thus, as an ATMAX2 homologous protein, the up-regulation of GRMZM2G393272_P02 at stages I and III in 18R but not in B73 imply its function in meristem development (Table 2). Additionally, GRMZM2G109284_P01, a SART-1 family protein, sharply increased at all 3 stages in 18R (Table 2); however, no changes were detected in B73. Arabidopsis *MDF* (belonging to SART-1 family protein), function in stem cell formation and meristematic activity^29^. The *mdf* mutant lost stem cells and lacked meristematic activity both in the root and vegetative shoot. Thus, the differences in expressions of GRMZM2G109284_P01 between 18R and B73 might be responsible for promoting callus (or stem cell) formation. Besides, Arabidopsis *ATCHITIV*, encoding an EP3 chitinase, was expressed in the cells surrounding the somatic embryo during somatic embryogenesis^30^. In the present study, GRMZM2G129189_P01, a gene homologous to *ATCHITIV*, was significantly up-regulated at all stages in 18R. However, it was increased only at stage III in B73 (Table 2). So, this gene might also play an important role in somatic embryogenesis.

The expressions of *AtFLA11* and *AtFLA12* were positively related to secondary cell-wall cellulose synthases in stems^31^. A homologous gene of *AtFLA11*, GRMZM2G022931_P01, increased sharply at stage II and stage III in both 18R and B73, whereas the fold change in 18R was higher than in B73 (Table 2), which might function in cell-wall cellulose synthases. During cotyledon callus induction, the outer cell wall was degraded. New cell walls were re-synthesized and cell wall synthesis related proteins were also reduced^16^. Cell wall loosening and/or remodeling is a necessary foundation for cell elongation and might be regulated by various hormones^32^.

It was reported that 14-3-3 proteins bind to pS/pT motifs to regulate several signal transduction pathways, such as ethylene^33^ and brassinolide^34^. In Arabidopsis, 14-3-3 proteins interact with ethylene-overproducer1 (ETO1)/ETO1-like (EOLs) to rapidly degrade 1-aminocyclopropane-1-carboxylate synthase^33^ to inhibit ethylene synthesis. As a 14-3-3 like protein, GRMZM2G408768_P01 was annotated as involving in cell cycle regulation and significantly increased at stage I in 18R and at all stages in B73 (Table 2), which might result in the down-regulation of ethylene (Fig. 6b) to further regulate the downstream genes (Fig. 4). Similarly, in the brassinolide transcription pathway, 14-3-3 proteins were found to bind to phosphorylated BZR1/BZR2, which efficiently retain proteins in the cytoplasm to further inhibit brassinolide levels^34^. Thus, GRMZM2G408768_P01 might act as a rapid response factor to ethylene and brassinolide synthesis at stage I to regulate the embryo expansion by controlling cell cycles.

Both proteomic and metabolomic analyses (Figs 3 and 4, Table 3, Supplementary Table S2) indicated that hormone synthesis and transduction, including auxin, cytokinin and brassinosteroids, play a role in EC induction. Combined with our previous study, the transcription levels of auxin influx carrier *AUX1*, auxin receptor *TIR1*, auxin-responsive protein *IAA*, *ARF* (auxin response factor) and *GH3* (auxin responsive *GH3* gene family) significantly changed during 18R EC induction^13^, which implies that the auxin transduction pathways were activated. Moreover, one *GH3* protein (GRMZM2G410567_P01) was sharply upregulated at all 3 stages in both maize lines (Fig. 5b). In addition, proteins expressed in 18R showed a higher positive correlation with auxin induction than B73 (Fig. 4). Altogether, 18R showed a stronger capability of auxin synthesis and transduction when cultured on auxin-containing medium.

It is widely accepted that the balance of auxin and cytokinin content in the cell is critical for callus induction in different plants^35^. Auxin is involved in cell proliferation and elongation, whereas cytokinin promotes cell proliferation and differentiation. The cytokinin transduction pathway was also activated during EC induction of 18R^13^. The present study indicated that the production of zeatin and dihydro-zeatin (cytokinin) was increased in 18R but without a change in B73 (Fig. 7a and b). Taken together, these findings suggest that the cytokinin synthesis in 18R is more critical for EC induction than in B73.

In response to EC induction, the synthesis of jasmonic acid was enahnced in 18R compared to B73 (Fig. 6). Furthermore, the proteins expressed during callus formation showed a significantly positive correlation with the treatment of jasmonic acid, which suggests that these proteins are regulated by jasmonic acid. Jasmonic acid is involved in biotic and abiotic stress responses, and plays especially a key role in the transduction of wound signals^36^. In addition to auxin, wound signals are generally considered to be another signal for callus induction^37^. A previous study showed that adding jasmonates to the medium could improve the callus formation ratio of *Vigna mungo* ^38^. Combined with the findings in the present study, the synthesis and transduction of jasmonic acid in 18R seems to partially contribute to its higher EC formation ratio than B73.

Based on discussed above, we hypothesized an EC induction mechanism (Supplementary Fig. S3). In summary, maize inbred line 18R and B73 show different stress response, energy supply, stem cell initiation, stem cell development, somatic embryogenesis, hormone synthesis, hormone transduction and hormone response abilities. All of these differences may be responsible for the high EC induction ratio of 18R.

## Methods

### Plant materials and tissue culture conditions

Maize inbred lines 18R and B73 were planted in a greenhouse (14/10 h light/dark, at 28 °C and 70% relative humidity). Immature embryos were harvested on the twelfth day after self-pollination. Modified N6 medium^13^ was used to induce embryonic callus, and the embryos were then incubated for 15 days in darkness at 28 °C. Immature embryos induced for 0 d were used as the control (CK). During 0–15 d, 500 mg calli were sampled every 24 h in 9 biological replicates (3 for protein extraction and 6 for metabolite isolation), quickly frozen in liquid nitrogen and stored at −80 °C.

### Metabolome analysis

The low-molecular-weight metabolites (<1000 Da) were isolated according to Decourcelle *et al*.^39^. Briefly, 100 mg of embryos or calli were ground to a powder in liquid nitrogen. Then, 1 ml of methanol (HPLC grade) was added and incubated at 70 °C for 15 min, followed by centrifugation for 10 min at 14,000 *g*. Finally, the supernatant was harvested. The metabolites from 1–5 d were equally mixed to form sample stage I (biological replicates 6 times); likewise, stage II (6–10 d), stage III (11 to 15 d) and CK were prepared. Then, 10 μL of these samples were submitted to HPLC-MS analysis. The MS data were analyzed using XCMS and CAMERA software^40^. To obtain high quality data, robust Loess signal correction (R-LSC)^41^ was used to normalize peaks to the QC samples, and the relative standard deviation (RSD) was calculated. The m/z with RSD value between 0 and 30% was subjected to principal component analysis (PCA), partial least-squares discriminant analysis (PLS-DA) and variable importance in the projection (VIP) analysis. Finally, the m/z with VIP > 1, fold change >1.5 (or <0.67) and Q value < 0.05 in any sample was retained for further metabolite identification and pathway annotation using the online HMDB database (http://www.hmdb.ca/), METLIN (http://metlin.scripps.edu/) and KEGG (www.genome.jp/kegg/) based on the differences between observed and theoretical mass smaller than 10 ppm.

### Protein extraction, iTRAQ labeling and LC-ESI-MS/MS analysis

Total protein was extracted as previously reported^42^. Then, 20 μg of the total proteins from 1 to 5 d was mixed to form samples of stage I (biological replicates 3 times); likewise, stage II (6–10 d), stage III (11 to 15 d) and CK (0 d) were prepared. Then, each mixed sample was digested with Trypsin Gold (Promega, Madison, WI, USA) at 37 °C for 16 hours and labeled according to the manufacturer’s protocol for the 8-plex iTRAQ reagent (Applied Biosystems). Four milliliters of buffer A (25 mM NaH2PO4 in 25% ACN, pH 2.7) was added to reconstitute the iTRAQ labeled peptide. Then, the peptides were eluted on a LC-20AB HPLC Pump system (Shimadzu, Kyoto, Japan) with a 4.6 × 250 mm Ultremex SCX column at a flow rate of 1 mL/min with buffer A for 10 min, 5–60% buffer B (25 mM NaH2PO4, 1 M KCl in 25% ACN, pH 2.7) for 27 min, 60–100% buffer B for 1 min, 100% buffer B for 1 min and buffer A for 10 min. The absorbance of the eluent was measured at 214 nm, and 20 fractions were obtained, desalted and vacuum-dried for further MS analysis.

Each fraction was re-suspended in buffer A (2% ACN, 0.1% FA) and centrifuged at 20,000 × *g* for 10 min. The supernatant was loaded onto the LC-20AD nanoHPLC (Shimadzu, Japan) and then separated at 300 nL/min starting from 2 to 35% B (98% ACN, 0.1% FA), followed by a 2 min linear gradient to 80%, 4 min at 80% B and finally returning to 5% in 1 min. After nanoelectrospray ionization, the peptides were submitted to tandem mass spectrometry (MS/MS) in the QEXACTIVE (Thermo Fisher Scientific, San Jose, CA) coupled online to the HPLC, followed by detection in the Orbitrap. High-energy collision dissociation (HCD) of the MS/MS was used to select peptides with a normalized collision energy setting of 27.0; ion fragments were detected in the Orbitrap at a resolution of 17,500. A data-dependent procedure that alternated between one MS scan followed by 15MS/MS scans was applied for the 15 most abundant precursor ions above a threshold ion count of 20,000 in the MS survey scan with a following Dynamic Exclusion duration of 15 s. An electrospray voltage of 1.6 kV was applied. Automatic gain control (AGC) was used to optimize the spectra generated by the Orbitrap. The AGC target for full MS was 3e6 and 1e5 for MS2. For MS scans, the m/z scan range was 350 to 2,000 Da. For MS2 scans, the m/z scan range was 100–1,800.

### iTRAQ protein identification and quantification

MS/MS data were searched using Mascot (2.3.02 version, Matrix Science, London, United Kingdom) against the Gramene database (Zea_maize, 105722 sequences) (ftp://ftp.gramene.org/pub/gramene/release42/data/fasta/zea_mays/pep/) to identify proteins. The search parameters were: mass tolerance of 20 ppm was permitted for intact peptide masses and 0.05 Da for fragmented ions; one missed cleavage was permitted for the trypsin digests; the potential variable modifications was Gln->pyro-Glu (N-term Q), oxidation (M) and deamidated (NQ), and fixed modifications were carbamidomethyl (C), iTRAQ8plex (N-term) and iTRAQ8plex (K); the charge states of peptides were set to +2 and +3; only peptides with Mascot probability greater than “identity” (95% confidence) were counted as identified. For protein quantitation, the proteins with at least two unique spectra in at least two replicates were kept for further analysis. The quantitative protein ratios were weighed and normalized by the median ratio in Mascot. Only the ratios with p-values < 0.05 and fold changes >1.5 in the 3 replicates were considered to be significantly changed^43^. The proteins that significantly changed in any inbred line or in any samples were selected for further analysis.

### Weighted gene co-expression network analysis

All of the selected proteins from 18R and B73 were independently subjected to signed hybrid weighted correlation network analysis through the R package WGCNA^14^ as previously reported^44^, using publically available scripts (http://labs.genetics.ucla.edu/horvath/CoexpressionNetwork/Rpackages/WGCNA/). Briefly, pairwise correlation matrices based on Pearson correlations between all pairs of proteins were constructed. Next, the power β of 12 (soft-threshold of the correlation matrix) was used to construct an adjacency matrix to raise the correlation matrix and suppress noisy small correlations. Based on the results of the adjacency matrix, the topological overlap was calculated to measure the network connections of gene pairs. According to the topological overlap, hierarchical trees with average linkage were built and cut with the Dynamic Tree Cut algorithm to group genes with highly similar co-expression relationships into the same modules. The first principal component (module eigengenes) of each module was used to represent its expression level.

### Module preservation analysis

To determine whether our modules were conserved, 20 permutations of the original data were subjected to module preservation statistical analysis to calculate *Zsummary* scores using the R function “modulePreservation” in the WGCNA package^14^. Then, the averaged *Zsummary* scores combined with SD (20 replicates) were used to summarize whether a module was preserved or not. As argued in the previous study, modules with *Zsummary* scores of >10 are strongly preserved. On the contrary, if *Zsummary* scores of <2, the modules are not preserved. *Zsummary* scores between 2 and 10 represent modules that are moderately preserved.

### Identifying differentially expressed proteins between 18R and B73

To identify the DEPs between 18R and B73, the following two methods were used. First, the significantly changed proteins of B73 were used to calculate the “module preservation” of 18R as illustrated above. The *Zsummary* scores here served as a measure to which each of the proteins of B73 was preserved in the 18R network. The modules with *Zsummary* scores below 2 were regarded as different modules; correspondingly, the proteins in these modules were identified as the DEPs between the two samples.

Secondly, we related B73 modules to 18R modules using WGCNA to obtain the correspondence of B73-specific and 18R-specific modules. Then, we used the eigengenes to represent the modules to calculate the Pearson correlations for each pair of modules between the two inbred lines. The corresponding proteins with correlations of <0.4 were also considered to be DEPs between 18R and B73.

### Gene ontology and pathway enrichment analysis

Blast2GO program (https://www.blast2go.com) was used against the non-redundant protein database (NR; NCBI) for functional annotations. The identified DEPs between 18R and B73 were subjected to Gene Ontology (GO) analysis using the web-based GO Enrichment Analysis tool (http://geneontology.org/). Pathway enrichment was analyzed using KOBAS 2.0 tools (http://kobas.cbi.pku.edu.cn/home.do).

### RNA isolation and quantitative real-time PCR

Total RNA was isolated using Trizol Reagent (Life Technologies, Carlsbad, CA, the United States). RNAs from 1–5 d (sample I) were mixed with equal proportion, as well as samples from 6–10 d (sample II) and 11–15 d (sample III). The RNAs extracted from 0 d were used as control. The reverse transcription was performed as the protocol of PrimeScript RT Reagent Kit with gDNA Eraser (Takara Biotechnology Co., Ltd., Dalian, China). The qRT-PCR was performed on ABI 7500 real-time PCR System (Applied Biosystems, USA). Actin 1 (GRMZM2G126010) was used as a reference gene. The primers for HDT2 (GRMZM2G100146) amplification are F:GATTCTGATGATTCTGATGAAGGCG and R: AGAAAGAGGCGTTTTCAGAGCAT. The primers for HDT3 (GRMZM2G159032) quantitative real time PCR are F:CGGACGATTCTGATGAGGGTT and R:TAGAGGAGTTTTCAGCACGGAAC.

## Electronic supplementary material

Supplemental Figures

Supplemental Tables
