# Supplementary material for: Metabolomic and Proteomic Analysis of Maize Embryonic Callus induced from immature embryo
Source: Sci Rep. 2017 Apr 21;7:1004. doi: 10.1038/s41598-017-01280-8 (PMC5430770; doi:10.1038/s41598-017-01280-8)
Supplement: Supplementary file 1 — Supplemental Figures [file 41598_2017_1280_MOESM1_ESM.pdf]

Metabolomic and Proteomic Analysis of Maize Embryonic Callus induced from immature embryo

Fei Ge[1+], Hongmei Hu[1+], Xing Huang[1], Yanling Zhang[1], Yanli Wang[1], Zhaoling Li[1], Chaoying Zou[1], Huanwei Peng [2], Lujiang Li[1], Shibin Gao[1], Guangtang Pan[1], Yaou Shen[1]*

[1] Key Laboratory of Biology and Genetic Improvement of Maize in Southwest Region, Maize Research Institute, Sichuan Agricultural University, Chengdu 611130, China;

[2] Institute of Animal Nutrition, Sichuan Agricultural University, Chengdu 611130, China.

[+] These authors contributed equally to this work.

* Correspondence author: Yaou Shen, shenyaou@aliyun.com.

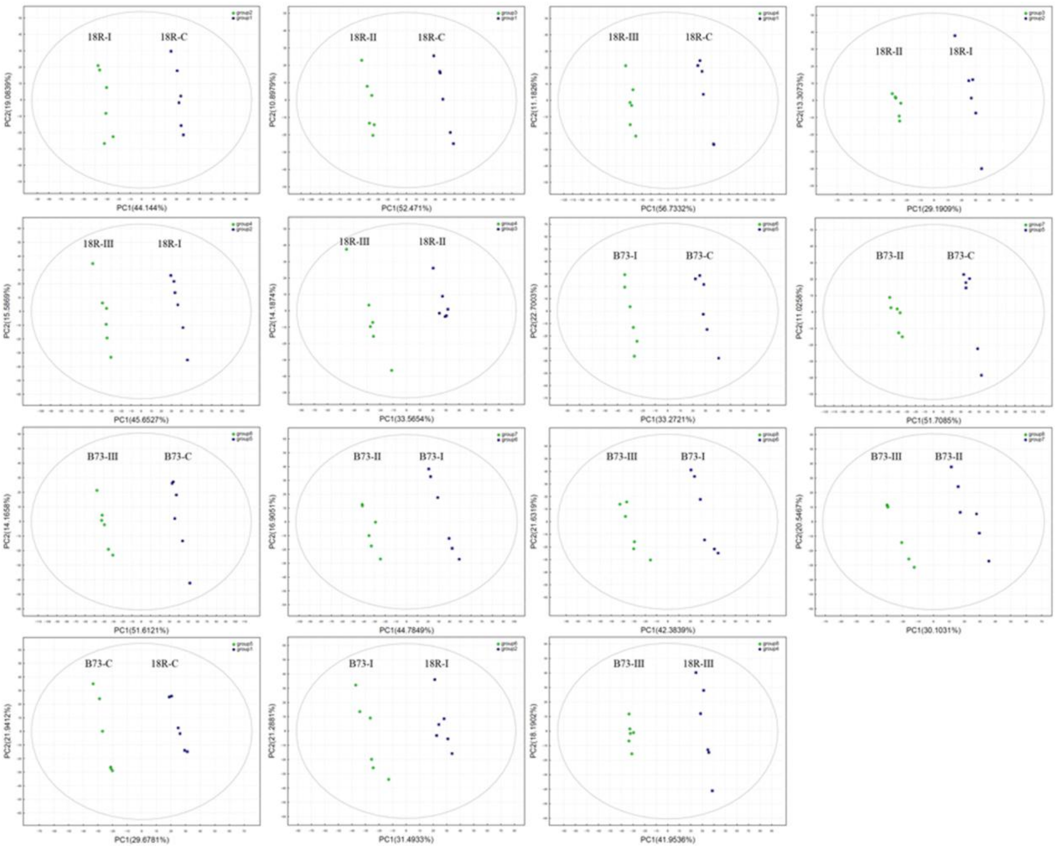

Supplementary Fig. S1. Partial least-squares discriminant analysis (PLS-DA) result of different stages in 18R and B73.

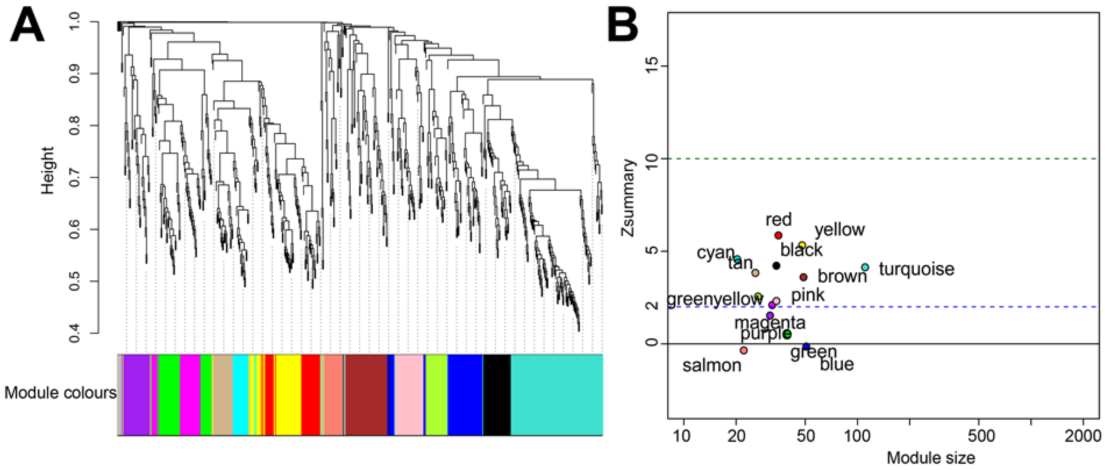

Supplementary Fig. S2. The modules of 18R (A) and module preservation results (B).

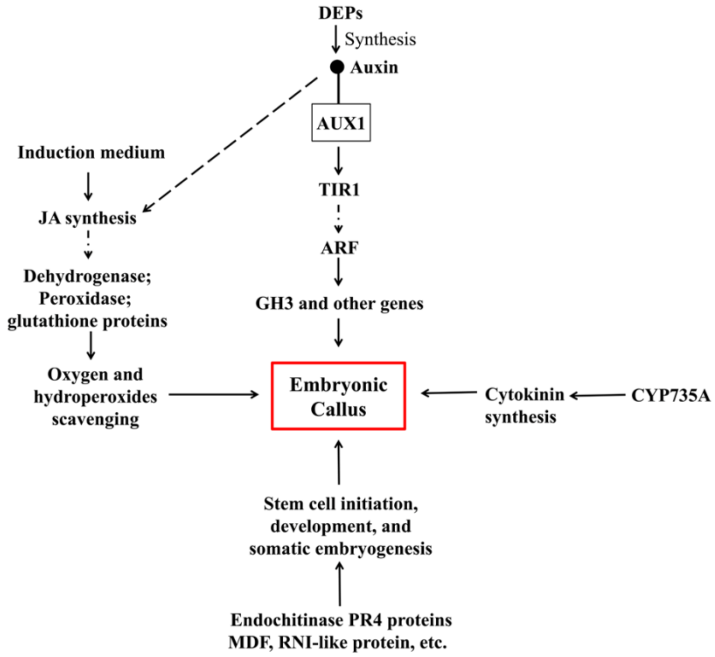

Supplementary Fig. S3. The hypothesized mechanism of EC induction.
